# Discordance Between Cross-Sectional Imaging and Pathology: A Rare Case of Xanthogranulomatous Inflammation of the Caecum and Appendix

**DOI:** 10.7759/cureus.109562

**Published:** 2026-05-24

**Authors:** Luke Thompson, Joshua Fultang, Rudi Schmigylski, Krsty Nale, Jozef Lastik, Karthik Maruthachalam

**Affiliations:** 1 General Surgery, Dumfries & Galloway Royal Infirmary, Dumfries, GBR; 2 Pathology, Dumfries & Galloway Royal Infirmary, Dumfries, GBR; 3 Radiology, Dumfries & Galloway Royal Infirmary, Dumfries, GBR

**Keywords:** colorectal disease, cross-sectional imaging, histopathology, right-sided hemicolectomy, xanthogranulomatous inflammation

## Abstract

Cross-sectional imaging of colonic pathology is usually correlated with other investigations, such as optical colonoscopy (OC), prior to surgical treatment. However, in acute inflammation, surgery may proceed based on imaging alone. This case describes a 78-year-old woman presenting with dyspnoea, fatigue, and weight loss. An unprepared computed tomography (CT) of the abdomen and pelvis demonstrated evidence of caecal wall thickening suggestive of malignancy. Colonoscopy was omitted in this case due to the possibility of perforation, and an elective right hemicolectomy was subsequently performed. Results on histopathology inspection revealed xanthogranulomatous inflammation (XGI) without dysplasia or malignancy. While CT, including CT colonography, plays an increasing role in colorectal cancer (CRC) diagnosis and assessment, differentiating malignancy from benign inflammation on cross-sectional imaging alone remains challenging, and OC with biopsy remains the diagnostic gold standard. Although rare, especially in the context of the caecum and appendix, XGI should be considered by clinicians, radiologists and pathologists as a differential diagnosis when CRC is suspected clinically and radiologically, without biopsy confirmation.

## Introduction

The limited accuracy of routine non-colonographic abdominal CTs in detecting colorectal cancers (CRCs) has been well documented in the literature [1-3]. Some studies have recorded sensitivities of as low as 75% whilst others report specificities ranging from 86% to 96% [2,3]. Suspicious colorectal lesions on unprepped CTs warrant further evaluation by luminal colonoscopy and biopsy prior to definitive management, especially in the absence of complications [4].

This report describes a patient who presented acutely with symptomatic anaemia and weight loss. An incidental finding of an abdominal mass in keeping with a primary caecal malignancy on CT, and subsequently, a right hemicolectomy was performed. Results on histopathology inspection revealed chronic xanthogranulomatous inflammation (XGI) of the caecum and appendix.

XGI is a rare benign chronic inflammatory pathology characterised by the presence of mixed inflammatory infiltrates and the accumulation of lipid-laden foamy macrophages [5]. It can be present in various organs, with those most commonly affected being related to the urinary tract or gallbladder. Even in commonly affected sites, XGI is rare (≈1.4 cases per 100,000 per year) [6], with colonic involvement even less frequent [7].

Our case report suggests that prior to histopathology confirmation, the differential diagnosis of XGI of the colon should be considered when CRC is suspected clinically and radiologically.

This article was previously presented as an abstract at the 2024 ASGBI Annual International Surgical Congress on May 10, 2024.

## Case presentation

A 78-year-old woman presented with progressive shortness of breath associated with fatigue and unintentional weight loss. She had no significant past medical history and no relevant family history. On physical examination, there was evidence of right basal crepitations and a palpable fixed right iliac fossa mass, which was non-tender on examination. At this acute initial presentation, the patient did not report any abdominal pain or bowel-related symptoms such as PR bleeding or altered bowel function.

**Figure 1:**
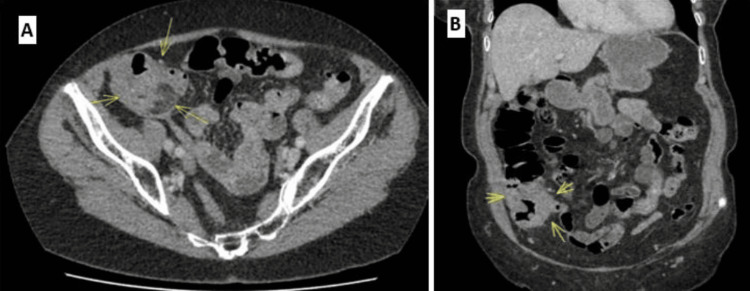
Contrast CT abdomen and pelvis (A) axial (B) coronal (area of suspicion marked by yellow arrows)

Initial bloods demonstrated a normocytic anaemia (Hb 105 g/L, MCV 86 fl), significantly raised D-dimer (3460 ng/mL) and raised C-reactive protein (133 mg/L) with normal white cell count (8.6x109/L). Subsequent computed tomography pulmonary angiogram (CTPA) and contrast CT abdomen and pelvis (CTAP) demonstrated extensive bowel wall thickening in the caecum with an appearance that was contiguous with a primary malignancy with suggestion of local extension and infiltration into the musculature of the anterior abdominal wall (Figures 1A-1B). The bowel wall demonstrated asymmetric circular thickening, more pronounced along the mesenteric border compared to the antimesenteric. Measured bowel wall thickness ranged from 8mm to 16mm. There was evidence of heterogeneous enhancement with hypo-attenuated areas and irregular margins noted at the base of the caecum, with associated adjacent fat stranding giving the impression of extramural extension. There was no cross-sectional evidence of low attenuation nodules. The presence of pericolic fat stranding was inferred to be in keeping with either extensive infiltration of the mesentery or a possible inflammatory reaction secondary to localised perforation. In regard to lymphadenopathy, a single sub-centimetre iliocaecal mesenteric lymph node was noted, with no suspicious features. There was no evidence of chest or liver metastases. Differential diagnosis, other than malignancy, included acute infection confined only to the caecum, while others, such as inflammatory bowel disease, were considered unlikely.

Following a colorectal multidisciplinary team (MDT) discussion, the decision was taken to proceed to an open right hemicolectomy without preoperative optical colonoscopy (OC). Suspicion of perforation on CT scan precluded the use of mechanical bowel preparation; hence, preoperative colonoscopy was not recommended. Since cross-sectional imaging also suggested the presence of muscle invasion, an open right hemicolectomy was planned in favour of a minimally invasive approach. An elective open right hemicolectomy was performed approximately five weeks after her index admission. Intraoperatively, there was evidence of partial lateral wall invasion, without muscle involvement. Her postoperative course was uneventful, and she was discharged on day six postoperatively.

**Figure 2:**
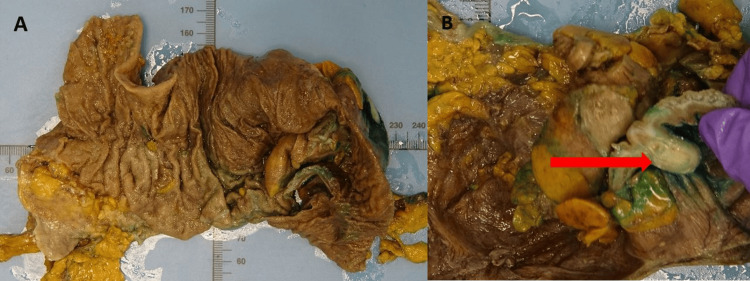
(A) Opened right hemicolectomy gross specimen demonstrating no evidence of caecal intraluminal lesion (appendiceal adhesions inked green); (B) section through the area of adhesion shows a focal lesion with a soft yellow-cream cut surface (marked with a red arrow)

Upon gross histopathological inspection of the specimen, the caecum showed no evidence of intraluminal malignancy. The appendix was adherent to the caecum with evidence of adhesions, which were inked green. On sectioning through this area, there was a suggestion of a mass lesion with the cut surface demonstrating a soft yellow-cream appearance (Figures 2A-2B).

**Figure 3:**
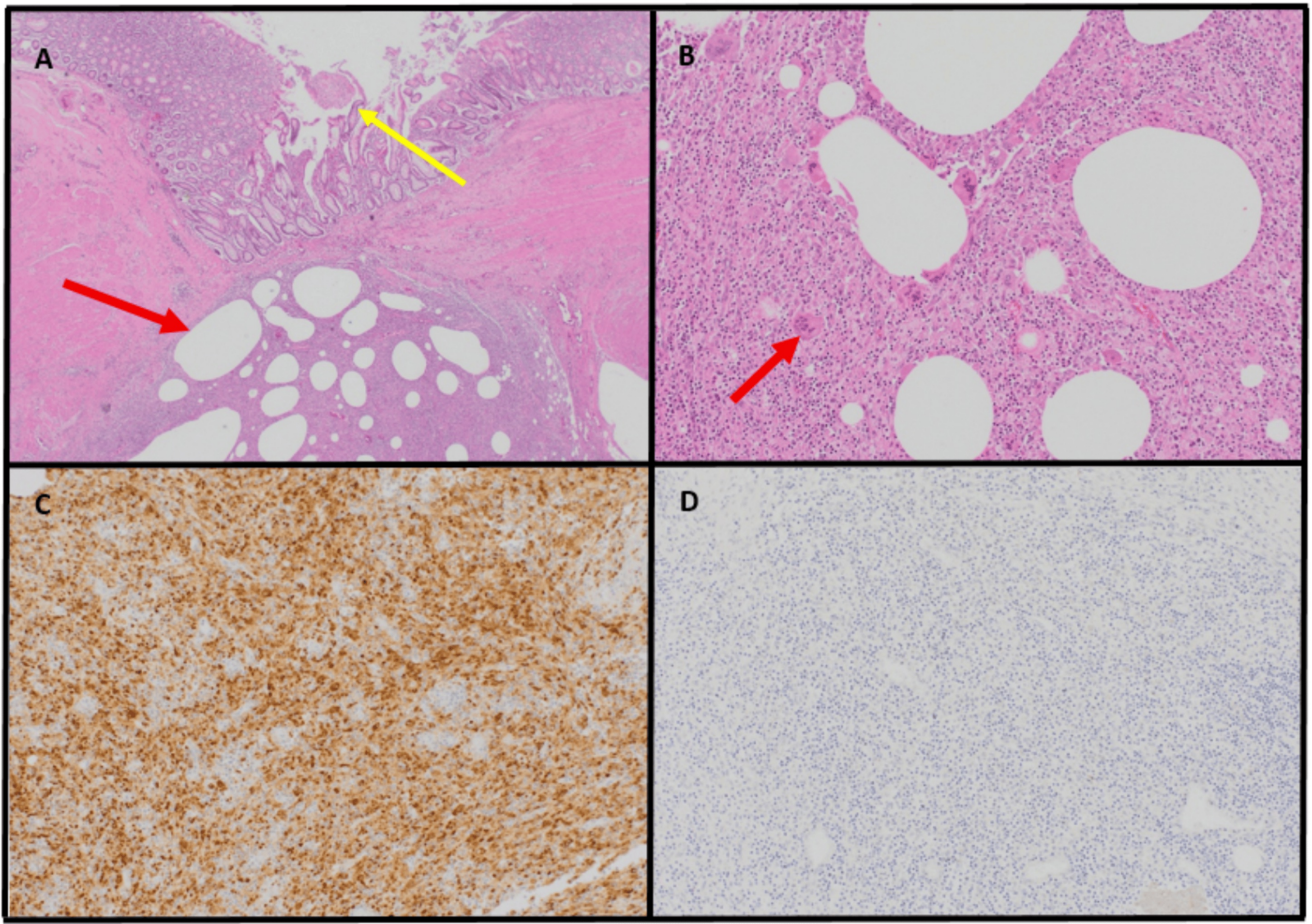
(A) Inflammatory submucosal mass (red arrow) with associated ulceration and inflammation of the overlying caecal mucosa (yellow arrow) (H&E, x20 magnification); (B) mass incorporates numerous foamy histiocytes and multinucleated giant cells (red arrow) in keeping with xanthogranulomatous inflammation (H&E, x100 magnification); (C) histiocytic differentiation confirmed by positive immunoreactivity with CD68 (CD68 stain, x100 magnification); (D) absence of staining with pancytokeratin rules out involvement by carcinoma (MNF 116 stain, x100 magnification) H&E: hematoxylin and eosin

Upon microscopic inspection of the caecal/appendiceal sections taken, the mass seen macroscopically was comprised of sheets of foamy histiocytes together with many multinucleated giant cells and mixed inflammatory cells in the background (Figures 3A-3B).

There was focal inflammation and ulceration of the caecal mucosa with no evidence of dysplasia or malignancy. Twenty-four regional lymph nodes retrieved from the specimen were also benign. Immunohistochemical analysis of this inflammatory mass showed positive expression with CD68, confirming histiocytic differentiation. Pancytokeratin markers were negative, ruling out poorly differentiated carcinoma in this case (Figures 3C-3D).

The patient was reviewed in the clinic six weeks post-right-hemicolectomy. On review, they were free of their original symptoms, which resulted in the initial presentation. Nine months postoperatively, an abdominal CT showed expected postsurgical changes with no features concerning of malignancy.

## Discussion

Tumours of the caecum constitute approximately 20% of all colorectal cancers (CRC), and often the presentation of such malignancies can be insidious due to the large lumen of the right colon [8]. Patients with caecal cancer may often present with a triad of non-specific signs that include right-sided pains, right-sided abdominal mass and microcytic anaemia. The standard diagnostic workup for patients with suspected CRC diagnosis includes endoscopic examination in the form of optical colonoscopy (OC) with biopsy for histopathological assessment, followed by computed tomography chest, abdomen and pelvis (CT CAP) to elicit local staging and evidence of metastatic disease [9]. The case we present is of an elderly patient who had convincing radiological evidence of a primary malignancy, who subsequently underwent colonic resection without the standardised endoscopic first approach.

Due to its high diagnostic performance, OC remains the gold standard and preferred investigation modality for the diagnosis of CRC [10]. Furthermore, the opportunity to obtain biopsy samples in patients with suspected CRC to attain a definitive diagnosis provides further evidence for the benefit of performing OC. However, technological advances in computer tomography (CT) and the use of virtual colonoscopy in the form of CT colonography have provided an alternative route for initial investigation and diagnosis of colorectal malignancy, particularly in those with borderline fitness or those unable to tolerate an OC, with the aim that a negative result can negate the requirement for more invasive investigation. The benefits of utilising this method include low risk of perforation, reduced discomfort and no requirement for sedation or recovery time when compared to an alternative such as OC [11]. When comparing the sensitivity for CRC detection, OC and CT colonography have been demonstrated to be relatively comparable, 94.7% and 96.1%, respectively [12]. Furthermore, when comparing patient experience of the two investigation modalities, the majority of patients find colonoscopy more unpleasant than CT colonography, 54% compared to 22% [11]. There is therefore evidence for the use of CT colonography, given its high sensitivity for CRC and higher patient acceptance, as an initial investigation in patients with suspected CRC. However, although not obligatory, it is still highly suggested that following positive CT colonography, patients should undergo examination with traditional OC for verification of results under direct visualisation and crucially, collection of biopsy specimens to ascertain a histopathological diagnosis [13]. However, approximately 30% of patients will present with a complication, such as obstruction or perforation, associated with their CRC as their first presentation and as such, urgent emergency colorectal surgery may be undertaken without diagnostic confirmation via colonoscopy and biopsy [14].

There is a significant overlap between imaging findings associated with CRC and benign colonic disease [15,16]. These mimics of CRC on CT include inflammatory bowel disease (IBD), diverticular disease, caecal ulcers or benign conditions of the appendix such as perforated appendicitis or mucocele. In the case of IBD and diverticular disease, common findings on CT can include symmetric or asymmetric bowel wall thickening, stenosis of the colonic lumen and pericolic fat stranding [16]. However, most bowel wall thickening seen on CT is nonspecific, has uncertain degrees of significance and could represent a normal variant [17]. The most common site for false positive errors in the diagnosis of CRC is within the sigmoid colon, as benign and inflammatory conditions such as diverticular disease are found most commonly within this section of large bowel; however, this is followed by the caecal territory, where appendiceal benign inflammatory masses are not uncommonly observed [3]. Given the often indistinguishable appearances of malignant and benign pathology on CT, this further outlines the benefits of performing diagnostic colonoscopy prior to resection surgery.

XGI is a rare entity that, as demonstrated in our case, can radiologically mimic colorectal malignancy by presenting as an infiltrative mass lesion. XGI was first reported in the genitourinary tract, but the involvement of various other organs has been described in the literature [18]. XGI is characterised by mixed inflammatory infiltrates and aggregation of lipid-laden foamy macrophages with the presence of foreign-body type or Touton giant cells [19]. The histological differential diagnosis includes malakoplakia, which may be distinguished by the presence of Von Kossa-positive Michaelis-Gutmann bodies [20]. In differentiating XGI from poorly differentiated carcinoma or diffusely infiltrating histiocytic lymphoma, immunohistochemical panels may be used as a diagnostic aid [21].

As described in this case report, XGI can mimic the appearance of CRC on CT imaging, with both often presenting as focal mass lesions with extension and inflammation to surrounding tissues, with or without lymph node involvement. Similar cases have been described within the hepatobiliary system, where xanthogranulomatous cholecystitis has mimicked the appearance of gallbladder cancer. However, subtle differences in imaging findings, such as increased diffuseness of wall thickening and submucosal hypo-attenuated nodules, have been observed more often in XGI but not within malignant gallbladders [22]. Similar cross-sectional imaging findings have been anecdotally noted in XGI of the gastrointestinal tract and, as such, may aid in the diagnosis of this rare benign inflammatory condition within the colon [23].

## Conclusions

There continues to be an emerging role for the use of CT imaging, especially CT colonography, in the investigation and diagnosis of CRC. However, the difficulty in distinguishing between malignant and non-malignant inflammation on CT further endorses the quintessential place for the use of OC with biopsy as the definitive investigation for patients with suspected CRC on cross-sectional imaging. Furthermore, OC with biopsy plays an important role in improving the understanding of both pathological and asymptomatic benign inflammation of the caecum. Although rare, this case report highlights that XGI should be considered by clinicians, radiologists and pathologists as a differential diagnosis when CRC is suspected clinically and radiologically, without biopsy confirmation.

## References

[1] Ozel B, Pickhardt PJ, Kim DH, Schumacher C, Bhargava N, Winter TC (2010). Accuracy of routine nontargeted CT without colonography technique for the detection of large colorectal polyps and cancer. Dis Colon Rectum.

[2] Ng CS, Doyle TC, Pinto EM (2002). Evaluation of CT in identifying colorectal carcinoma in the frail and disabled patient. Eur Radiol.

[3] Ganeshan A, Upponi S, Uberoi R, D'Costa H, Picking C, Bungay H (2007). Minimal-preparation CT colon in detection of colonic cancer, the Oxford experience. Age Ageing.

[4] Mangat S, Kozoriz MG, Bicknell S, Spielmann A (2018). The accuracy of colorectal cancer detection by computed tomography in the unprepared large bowel in a community-based hospital. Can Assoc Radiol J.

[5] Cozzutto C, Carbone A (1988). The xanthogranulomatous process. Xanthogranulomatous inflammation. Pathol Res Pract.

[6] Harley F, Wei G, O’Callaghan M (2023). Xanthogranulomatous pyelonephritis: a systematic review of treatment and mortality in more than 1000 cases. BJU International.

[7] Yoon JS, Jeon YC, Kim TY (2013). Xanthogranulomatous inflammation in terminal ileum presenting as an appendiceal mass: case report and review of the literature. Clin Endosc.

[8] Hermann J, Karmelita-Katulska K, Paszkowski J, Drews M, Stajgis M (2011). Diagnosis of a cecal tumour with virtual colonoscopy. Pol J Radiol.

[9] Scottish Intercollegiate Guidelines Network (2025). Diagnosis and management of colorectal cancer: a national clinical guideline. Diagnosis and management of colorectal cancer.

[10] Kekelidze M, D'Errico L, Pansini M, Tyndall A, Hohmann J (2013). Colorectal cancer: current imaging methods and future perspectives for the diagnosis, staging and therapeutic response evaluation. World J Gastroenterol.

[11] Schima W, Mang T (2004). CT colonography in cancer detection: methods and results. Cancer Imaging.

[12] Pickhardt PJ, Hassan C, Halligan S, Marmo R (2011). Colorectal cancer: CT colonography and colonoscopy for detection--systematic review and meta-analysis. Radiology.

[13] Barish MA, Soto JA, Ferrucci JT (2005). Consensus on current clinical practice of virtual colonoscopy. AJR Am J Roentgenol.

[14] Elmessiry MM, Mohamed EA (2020). Emergency curative resection of colorectal cancer, do it with caution. A comparative case series. Ann Med Surg (Lond).

[15] Ridereau-Zins C (2014). Imaging in colonic cancer. Diagn Interv Imaging.

[16] Ganguly A, Meredith S, Probert C, Kraecevic J, Anosike C (2016). Colorectal cancer mimics: a review of the usual suspects with pathology correlation. Abdom Radiol (NY).

[17] Iadicola D, De Marco P, Bonventre S, Grutta EM, Barletta G, Licari L, Gulotta G (2018). Bowel wall thickening: inquire or not inquire? Our guidelines. G Chir.

[18] Kang TW, Lee KH, Piao CZ (2007). Three cases of xanthogranulomatous epididymitis caused by E. coli. J Infect.

[19] Dhawan S, Jain D, Kalhan SK (2011). Xanthogranulomatous inflammation of ascending colon with mucosal involvement: report of a first case. J Crohns Colitis.

[20] Grunhut J, Oroz R, Brown S, Nazarian-Rostami R (2022). Renal malakoplakia with invasion of the liver and diaphragm: a patient case and literature review. BMJ Case Rep.

[21] Krishna M, Dayal S (2021). Xanthogranulomatus inflammatory lesion mimicker of malignancy: a clinicopathological study from rural India. North Clin Istanb.

[22] Rommohan A, Cherukuri SD, Sathyanesan J, Palaniappan R, Govindan M (2014). Xanthogranulomatous cholecystitis masquerading as gallbladder cancer: can it be diagnosed preoperatively?. Gastroenterol Res Pract.

[23] Kapoor A, Soni D, Paramanadhan M, Kini L, Beniwal S, Kumar H (2015). Xanthogranulomatous Colitis masquerading as carcinoma of colon. Int J Cancer Ther Oncol.

